# Pulmonary Arterial Aneurysms and Thrombosis in a Young Male: A Rare Presentation of Behcet's Disease

**DOI:** 10.7759/cureus.41928

**Published:** 2023-07-15

**Authors:** Bapi Raju V Kurada, Chukwuemeka A Umeh, Rakesh C Gupta, Stella C Onyi, Jose Penaherrera

**Affiliations:** 1 Internal Medicine, Hemet Global Medical Center, Hemet, USA; 2 Medicine, St. George's University School of Medicine, Grenada, GRD; 3 Pulmonary and Critical Care Medicine, Hemet Global Medical Center, Hemet, USA; 4 Radiology, Hemet Global Medical Center, Hemet, USA

**Keywords:** deep vein thrombosis (dvt), behcet's disease, vasculitis, pulmonary artery thrombosis, pulmonary artery aneurysm

## Abstract

Behcet's disease (BD) is a chronic systemic inflammatory vasculitis with a relapsing and remitting course. The disease predominantly affects males between the ages of 20 and 40 years. The disease is more prevalent in Middle Eastern and Asian countries but is less common in North American countries. BD typically presents as recurrent oro-genital ulcers and ocular inflammation. Pulmonary vasculitis with pulmonary arterial involvement is a unique manifestation, with most pulmonary manifestations occurring later in the disease course. Here, we report a case with pulmonary arterial aneurysms and variable arterial-venous thrombosis in a young African American Male diagnosed with BD after he presented with pulmonary manifestations. This report emphasizes that a high index of suspicion is needed to diagnose a rare condition with such variable manifestations as Behcet's disease and that early detection and immunosuppression therapy can confer improved prognosis.

## Introduction

Behcet's disease (BD) is an inflammatory vasculitis with multiorgan involvement. The exact etiology of the disease is unknown. It is thought to be due to a chronic relapsing and remitting course. The current hypothesis indicates that BD mostly affects young adults, with preponderance in males, who also tend to exhibit more severe disease [1,2]. The mean age of occurrence is between 20 and 40 [3,4] though cases have been reported as early as pre-puberty until the fifth decade [5]. Traditionally described as the "silk road disease," BD has the highest prevalence in Turkey, with 80 to 420 cases per 100,000 persons [5], followed by Iran and Japan [6]. The prevalence in Western countries is low, where BD is considered a rare disease with a prevalence ranging between 0.12-0.64 per 100,000 persons [5].

The disease causes recurrent oro-genital ulcers and uveitis. Less common sites include cutaneous, articular, vascular, pulmonary, and gastrointestinal systems, leading to significant morbidity and mortality. Vasculitis in BD affects both arteries and veins of various sizes [1,2], unlike other types of vasculitis. Hence, the disease is classified as a "variable vessel vasculitis" by the Revised International Chapel Hill Consensus Conference Nomenclature of Vasculitides [7]. Within the arterial bed, aneurysms are more frequent than thrombosis. Among different organ systems affected, pulmonary vascular involvement is rare [3], with pulmonary arterial thrombosis (PAT) considered even more uncommon [1]. Here, we report a rare case that presented to our emergency department with repeated episodes of hemoptysis, pulmonary arterial aneurysm, pulmonary arterial thrombosis, and deep vein thrombosis (DVT).

## Case presentation

The patient was a 24-year-old African American male with a history of left lower extremity DVT on Eliquis who presented to our emergency department with recurrent episodes of hemoptysis. Other associated symptoms included cough and worsening shortness of breath on exertion, 20 lbs unintentional weight loss over the past six months, recurrent aphthous ulcers, and enlarged left scrotum. He denied visual abnormalities, skin lesions, and testicular/scrotal/penile ulcers. He denied current or recent viral illness or drug use. He worked as an auto mechanic until recently. Family history is positive for DVT in a paternal uncle and negative for cancers, vasculitis, or autoimmune pathology. Vital signs were normal. Clinical examination revealed a tender aphthous ulcer on the mucosal aspect of the upper lip and left non-tender scrotal enlargement with dilated left scrotal veins but otherwise unremarkable.

Laboratory analysis was significant for elevated erythrocyte sedimentation rate (ESR), high-sensitivity C-reactive protein (CRP), D-dimer, prothrombin time (PT), partial thromboplastin time (PTT), and international normalized ratio (INR) (Table 1). Urinalysis showed microscopic hematuria with 2-4 RBCs and otherwise unremarkable. Arterial blood gas (ABG) analysis indicated mild respiratory alkalosis with PH 7.48, partial pressure of carbon dioxide (PCO_2_) 32, partial pressure of oxygen (PO_2_) 145, bicarbonate (HCO_3_) 23.8, and O_2_ saturation 100% on room air.

**Table 1:** Laboratory values BUN: blood urea nitrogen; GFR: glomerular filtration rate; SARS-COV-2: severe acute respiratory syndrome coronavirus 2;

Investigations	Values	Reference range
Complete blood count		
Hemoglobin	13.4	(12.5-16.3) g/dL
Hematocrit	41.8	(36.7-47.1)%
Mean corpuscular volume	76.4 (L)	(80-97) fL
White blood cell count	6	(3.6-11.2) 10*3/mL
Red blood cell count	5.47	(4.06-5.63) 10*6/mL
Platelets	362	(140-400)Thousand/uL
Renal function tests		
BUN	18	(7-25) mg/dL
Creatinine	1.18	(0.7-1.3) mg/dL
GFR	93	mL
Electrolytes		
Sodium (Na^+^)	140	(135-145) mEq/L
Potassium (K^+^)	3.8	(3.5-5.1) mEq/L
Chloride (Cl^-^)	105	98-107) mEq/L
Thrombophilia profile		
Prothrombin time	15 (H)	(10.4-12.8) s
Partial thromboplastin time	39.2 (H)	(25.2-34.2) s
International normalized ratio (INR)	1.3 (H)	(0.9-1.1)
D-dimer	562 (H)	(<230 ng/mL)
Liver function tests		
Total protein	8.7	(6.4-8.9) g/dL
Aspartate transaminase	12 (L)	(13-39) U/L
Alanine transaminase	10	(7-52) U/L
Alkaline phosphatase	120 (H)	(34-104) IU/L
Lipid profile		
Total cholesterol	195	<200 mg/dL
Triglycerides	75	<150 mg/dL
High-density lipoprotein	40 (L)	>50 mg/dL
Low-density lipoprotein	140 (H)	<100 mg/dL
Viral markers and infectious workup		
Human immunodeficiency virus* *(HIV)	Non-reactive	Non-reactive
Adenovirus	Not detected	Not detected
Coronavirus strains	Not detected	Not detected
SARS-COV-2	Not detected	Not detected
Human metapneumovirus	Not detected	Not detected
Human Rhinovirus/Enterovirus	Not detected	Not detected
Influenza A	Not detected	Not detected
Influenza B	Not detected	Not detected
Para influenza 1-4	Not detected	Not detected
Respiratory syncytial virus	Not detected	Not detected
Bordetella pertussis	Not detected	Not detected
Bordetella parapertussis	Not detected	Not detected
Chlamydia pneumoniae	Not detected	Not detected
Mycoplasma pneumoniae	Not detected	Not detected
Other tests		
Erythrocyte sedimentation rate	120 (H)	0-14 mm
Highly sensitive C-reactive protein	0.21	<0.1 mg/dL
(L): Low; (H): High		

**Figure 1:**
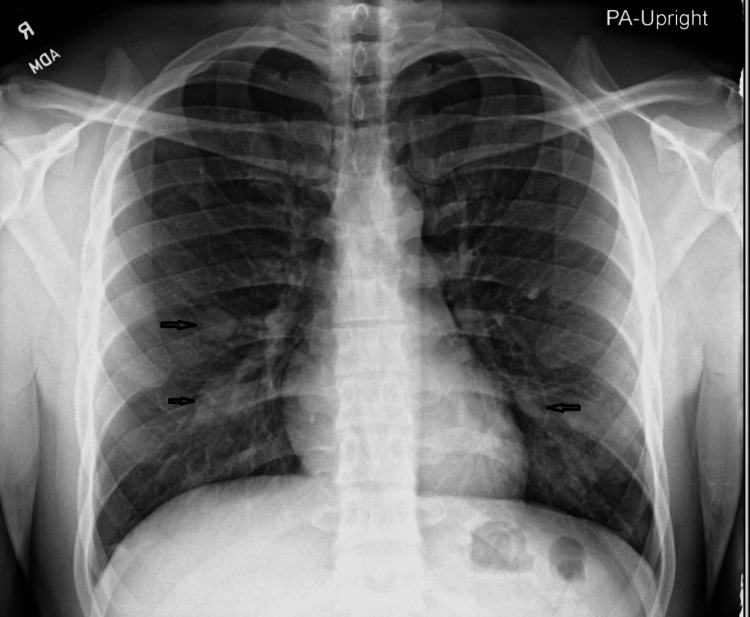
Right medial, middle lung zone, and bilateral medial lower lung zones nodular densities. (Black arrows)

Initial chest radiograph obtained at the presentation demonstrated nodular densities over the bilateral medial lower lung zones and right medial mid-lung zone (Figure 1).

**Figure 2:**
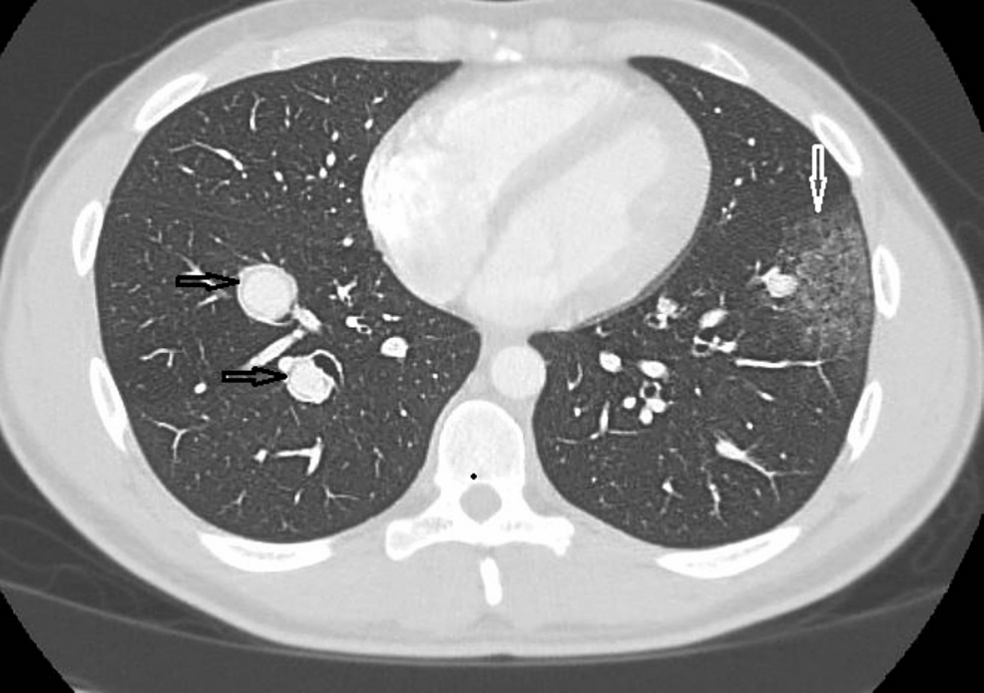
Right lower lobe enhancing nodular densities (black arrows) with left lower lobe patchy ground-glass opacity (white arrow).

A computerized tomography (CT) angiogram of the chest showed multiple enhancing nodular foci along the pulmonary arterial system suggestive of diffuse pulmonary aneurysms associated with focal left lower lobe ground glass density, indicating possible local hemorrhage (Figure 2). Eliquis was stopped, and the patient was admitted for further workup.

**Figure 3:**
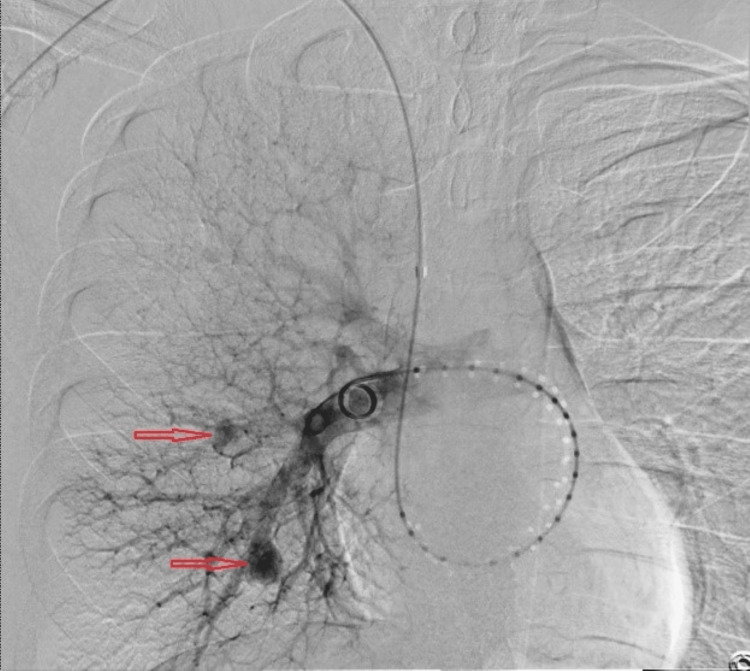
Right-sided pulmonary artery angiogram demonstrating the right-sided aneurysms (red arrows)

A pulmonary artery angiogram obtained demonstrated the pulmonary artery aneurysms (Figure 3).

**Figure 4:**
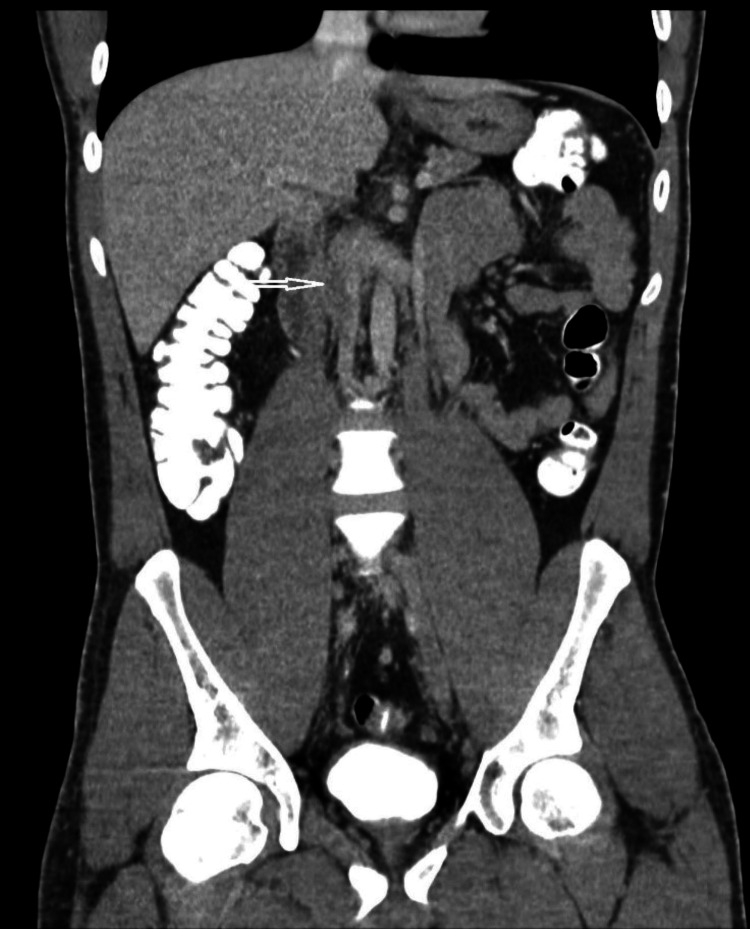
Thrombus within the infra-renal aorta (white arrow)

**Figure 5:**
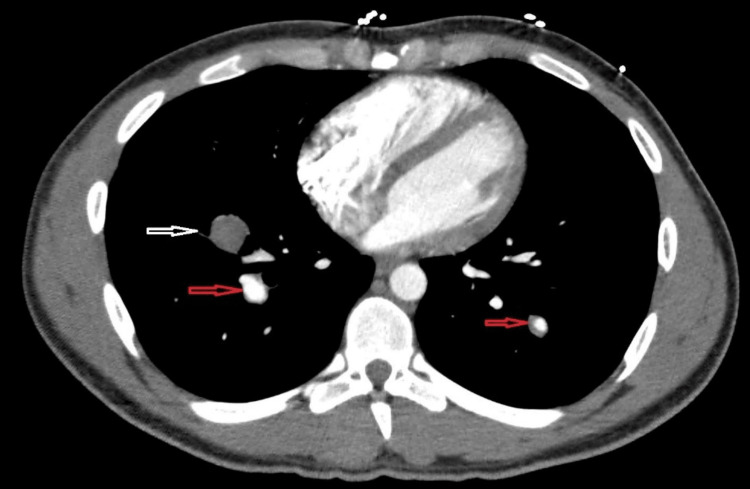
Completely thrombosed pulmonary artery aneurysm (white arrow) with partially thrombosed pulmonary aneurysms (red arrows)

**Figure 6:**
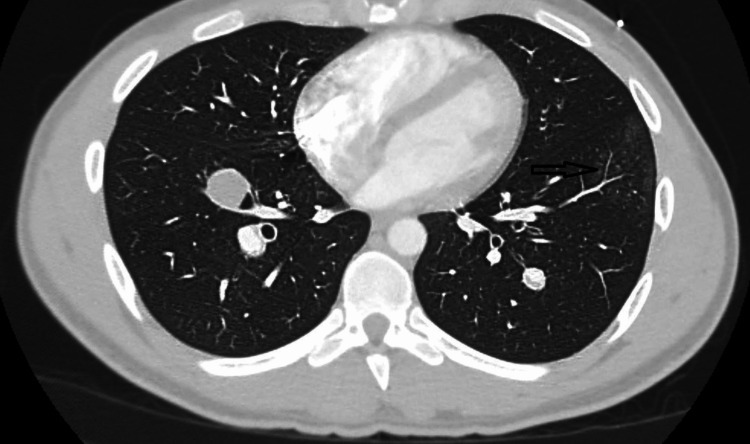
Improving right lower lobe patchy ground-glass opacity (black arrow)

A subsequent CT chest/abdomen/pelvis with intravenous and oral contrast done three days later revealed extensive long-segment occlusion of the infrarenal segment of the inferior vena cava (IVC) as well as common iliac veins (large vessel vasculitis) (Figure 4), interval thrombosis and partial thrombosis of the pulmonary artery aneurysms (Figure 5), and interval improvement in the left lower lobe patchy ground-glass opacity (Figure 6).

**Figure 7:**
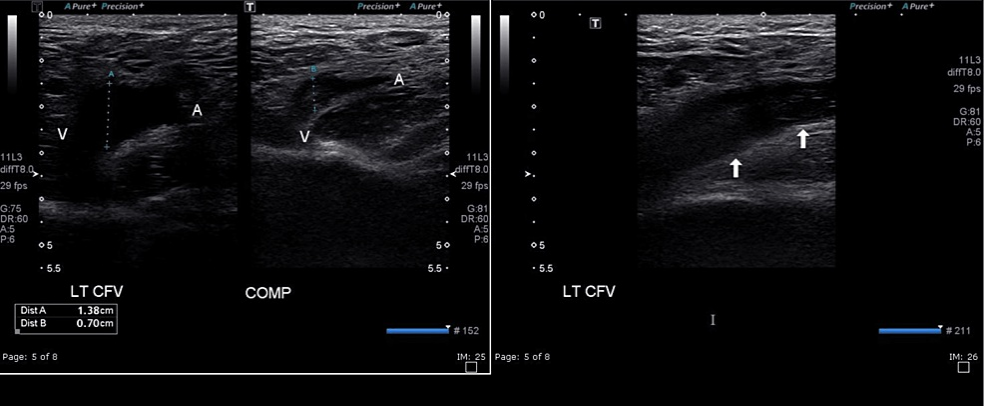
Non-compressibility of the left common femoral vein with visualized thrombus within the common femoral vein

Ultrasound (US) of bilateral lower extremity veins showed non-occlusive DVT in the left lower extremity (LLE) extending from the popliteal vein to the common femoral vein (medium vessel vasculitis) (Figure 7).

**Figure 8:**
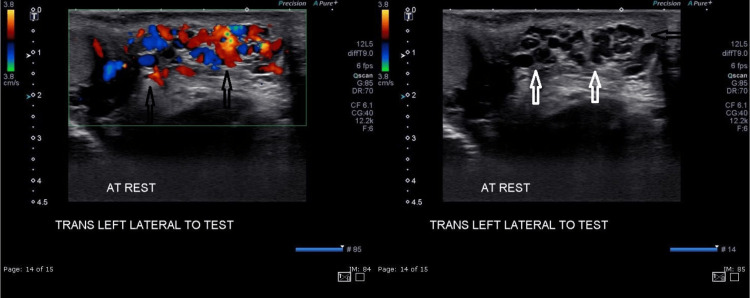
Anechoic area consistent with dilated pampiniform plexus (white arrows) with blood flow noted on color Doppler (black arrows)

US testicular sonogram showed left-sided varicocele with minimal hydrocele (small vessel vasculitis) (Figure 8).

After ruling out infectious etiology, septic emboli, and underlying coagulopathic disease, and based on the findings of multiple arterial aneurysms with venous thrombosis of large, medium, and small vessel thrombosis, a diagnosis in the presence of an aphthous ulcer was suspected to be Behcet's disease, which was later confirmed with a pathergy test. Incomplete Behcet's disease/Hughes-Stovin syndrome (HSS) was considered less likely due to the presence of aphthous ulcers, which are very rare in HSS [8,9]. Anticoagulation therapy was not initiated due to the high risk of bleeding. IVC filter was considered but not placed due to the chronicity of the thrombus. He was started on methylprednisolone 1 g IV daily for three days and then switched to oral prednisone 60 mg oral daily along with azathioprine 50 mg oral daily. At this time, the patient was hemodynamically stable due to 100% O_2_ saturation on room air, the ability to mobilize without distress, and the absence of further bleeding episodes. He was discharged home on prednisone and azathioprine combination therapy and recommended to follow up with a rheumatologist on an outpatient basis. The patient did follow up with a rheumatologist, and the outpatient pathergy test was positive, further confirming the diagnosis of Behcet's disease. The rheumatologist recommended HLA-B*51 genetic testing, but the patient did not do it due to out-of-pocket costs. The rheumatologist also suggested switching azathioprine to cyclophosphamide, but the patient refused due to the side-effect profile. The patient was without new bleeding episodes during his last visit to the outpatient clinic.

## Discussion

Clinical manifestations in BD typically involve the entire body but are most commonly characterized by the triple-symptom complex (recurrent oral aphthous ulcers, genital ulcers, and ocular lesions) [5]. Among uncommon presentations, pulmonary vascular disease is considered to be an unusual presentation characterized by pulmonary arterial aneurysms (PAA), pulmonary arterial thrombosis (PAT) (rarest presentation), and hemorrhage [1,2]. This is also a major contributor to morbidity and mortality in BD. There are no specific diagnostic tests or biomarkers to diagnose or assess the disease severity [5]. Diagnosis is mostly based on the clinical picture and is one of exclusion. The rarity in presentation demands a high index of suspicion for BD. Aphthous ulcers are common enough, but the concomitant presence of pulmonary arterial aneurysms and superimposed arteriovenous thrombosis should raise a red flag. There is a long delay in diagnosing BD because of the variable and intermittent presentation [2], leading to more severe presentations in Western countries [3]. This is likely the reason for the severe presentation in our patient. His initial presentation was DVT after a short two-hour flight, followed by recurrent bouts of hemoptysis after a few months. It has also been reported that earlier age of onset of BD is correlated to increased severity of presentation and mortality [5]. Vascular manifestations as thought to arise three to 16 years after the oral aphthous ulcers manifest [5]. It is unclear how long our patient has suffered from these oral ulcers. In addition, a 20 lb unintentional weight loss over the past six months was reported. It is possible that the disease process in our patient started at least three years ago, and his initial presentation to the hospital was with DVT after only a short flight. A high index of suspicion is necessary, and investigations are to be started in a patient who does not have any risk factors and presents with thrombosis only with minor triggers.

BD is the only vasculitides that cause pulmonary artery aneurysms, and pulmonary artery aneurysm is the leading cause of mortality in Behcet's syndrome [10]. Hemoptysis is the commonest presentation, with about 90% of the patients presenting with hemoptysis and 40% to 50% with massive hemoptysis (>500 cc) [10,11]. However, pulmonary artery aneurysms in Behcet's disease are rare, with prevalence rates of 0.6% to 1.4% of pulmonary artery aneurysms reported in patients with Behcet's disease [10,11]. Males are predominantly affected. Pulmonary artery aneurysms are strongly associated with thrombophlebitis, with more than 70% of patients with pulmonary aneurysms having coexistent lower extremity venous thrombosis [10,12]. This is similar to our patients who also presented with a DVT.

No curative treatment exists for BD, and treatment is based on the severity of clinical manifestation. Administering anticoagulants in BD is controversial. The exact etiology of vasculitis is unknown but is thought to be due to inflammation [1,13]. Hence, the treatment should be geared toward reducing inflammation. This can be achieved with high-dose corticosteroids and other immunosuppressant agents such as azathioprine and cyclophosphamide [1]. Furthermore, BD patients with pulmonary aneurysms are usually treated with cyclophosphamide for at least one year combined with prednisolone. Prednisolone can be started at three 1 g IV pulses and then oral 1 mg/kg per day dose. Tapering can begin at 10 mg/mo after the first month, and prednisolone is usually continued for at least one to two years at 5-10 mg/d [10,14,15]. After the induction with cyclophosphamide, patients can be maintained on another immunosuppressive therapy such as azathioprine, interferon, or colchicine [10,14,15]. Our patient was initially started on azathioprine and steroids because we did not have cyclophosphamide available in the hospital pharmacy then. Azathioprine was started at 50 mg orally daily concomitantly for titration as tolerated to 2.5 mg/kg body weight/day [16]. Other treatment modalities include embolization of the pulmonary aneurysm and surgery, including lobectomy for patients with massive or treatment-resistant hemoptysis, have been reported [10]. The role of anticoagulants in BD is limited due to the high risk of bleeding [13]. Our patient presented with recurrent bouts of hemoptysis following anticoagulation therapy. Additionally, hemoptysis could also result from rupture of pulmonary arterial aneurysms. Hence, Eliquis was stopped.

The prognosis of BD is unpredictable. Massive hemoptysis is the commonest cause of death. The mortality rate in patients with pulmonary aneurysms has improved over the years with treatment, with 62% to 71% ten-year survival rates reported [10,11]. However, fatal outcomes were more likely in patients with an aneurysm with the largest diameter ≥3 cm than those with a diameter <3 cm [11]. While arterial involvement significantly affects mortality [17,18], early detection and treatment with immunosuppressants might confer a better prognosis [1,3,18].

## Conclusions

Pulmonary artery aneurysm and thrombosis is an extremely rare presentation of BD but carries significant morbidity and mortality. The available literature on the efficacy of treatment modalities is mainly limited to case reports, case series, and a few systematic reviews. The management of BD has evolved with the availability of immunosuppressive agents; however, the success rates continue to stay slim. The current treatment strategy focuses on managing vasculitis in BD with immunosuppressant medications, while the role of anticoagulants is debatable. A lack of detailed understanding of the etiopathogenesis of BD makes the management challenging and the prognosis unpredictable. Large, multicenter randomized clinical trials are needed to further our knowledge of this complicated disease and its management.
